# Supplementary material for: Smell and taste changes are early indicators of the COVID-19 pandemic and political decision effectiveness
Source: Nat Commun. 2020 Oct 14;11:5152. doi: 10.1038/s41467-020-18963-y (PMC7560893; doi:10.1038/s41467-020-18963-y)
Supplement: Supplementary file 1 — Supplementary Informations [file 41467_2020_18963_MOESM1_ESM.pdf]

Supplementary Figures

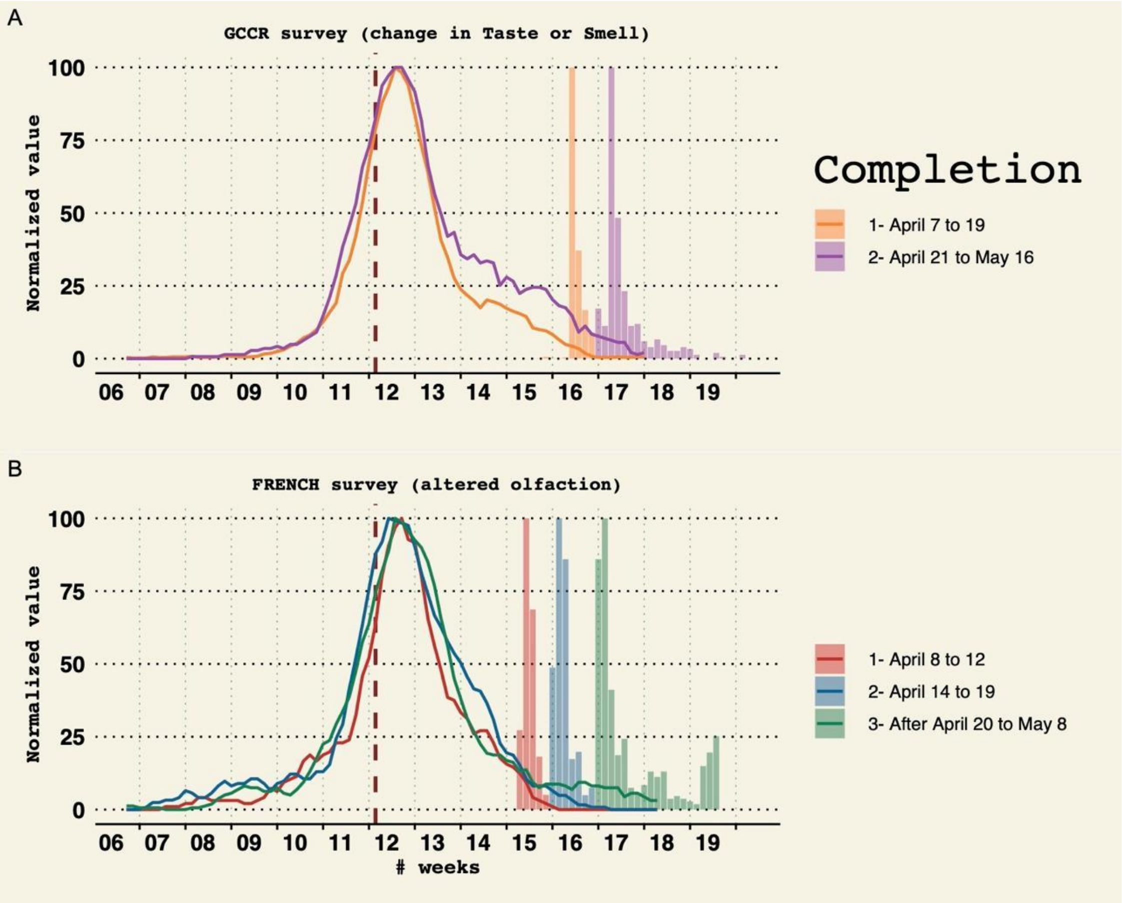

Supplementary Figure 1. *Evaluation of the robustness of reported smell and taste changes over time. Colored lines represent the proportion of reported onset of smell and taste change normalized to the highest value of each series. The barplot indicates the proportion of the date of completion of the survey (normalized to the highest value of each series). Colors represent groups of participants according to completion time. French lockdown is represented by the vertical brown dashed line.* ***A)*** *Peaks of smell/taste changes in the French participants who answered the GCCR questionnaire according to different dates of completion (before or after April 20).* ***B)*** *Date distribution of an independent French survey performed on 950 individuals and focusing on smell alterations in the French population independently of COVID-19 (see* ***Methods****).*

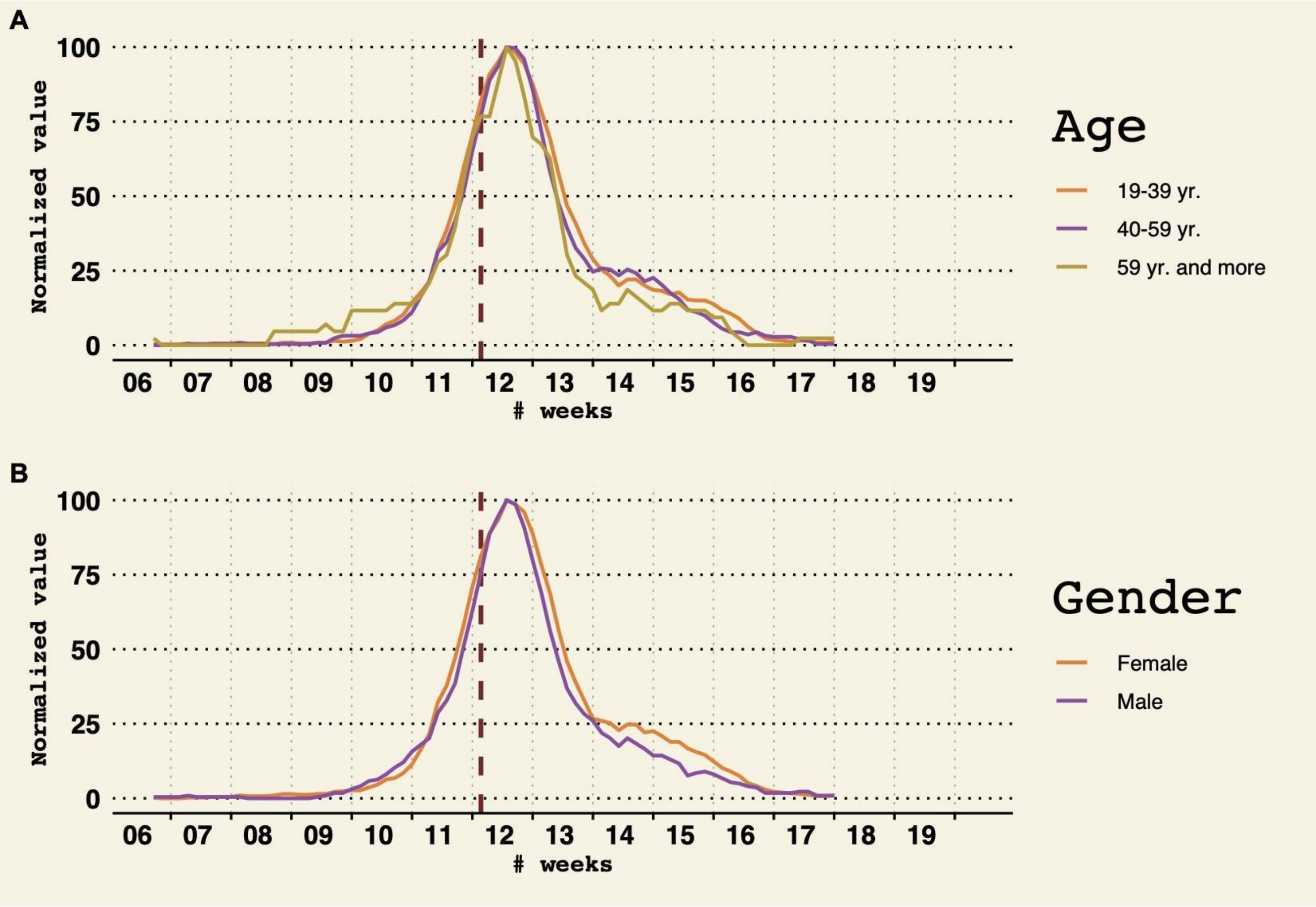

Supplementary Figure 2. *Time of peaks of smell/taste changes in French participants who answered the GCCR questionnaire according to their age or gender. Similarly to figure S1, colored lines represent the proportion of reported onset of smell and taste normalized to the highest value of each series. Colors represent groups of participants according to age (S2.A) or gender (S2.B).*

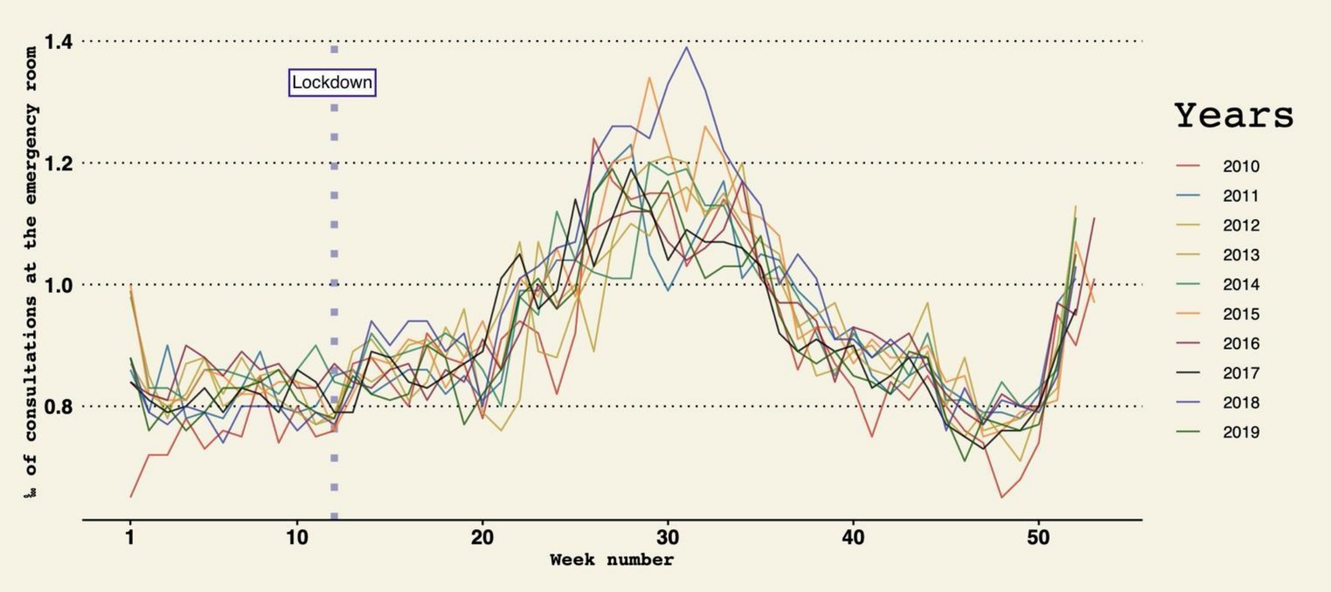

Supplementary Figure 3. *Evolution of the ratio of consultation for allergies in the emergency room (for 10.000 consultation) over time during the last 10 years. The start of the 2020 lockdown is represented by the vertical blue line.*

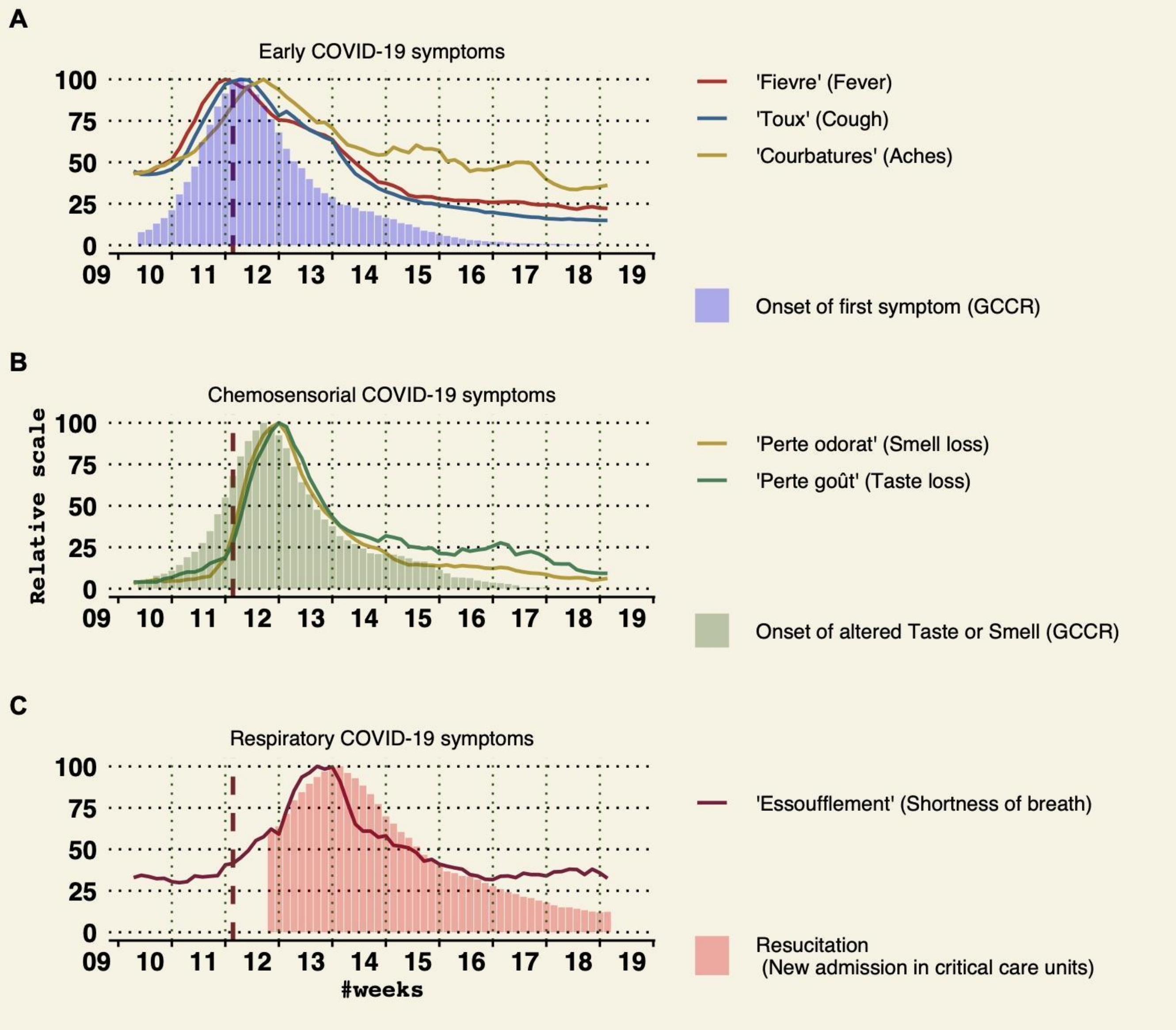

Supplementary Figure 4. *Google trends in France for COVID-19 symptoms. The French lockdown start is shown by a brown dashed line.* ***A)*** *The peak of the onset of first symptoms declared in the GCCR survey (blue bars, 1 day after the lockdown) corresponds to peaks of online queries for fever (red line), cough (blue line) and aches (yellow line).* ***B)*** *The peak of onset of altered smell/taste (green bars, 4 days after the lockdown) corresponds to peaks of online queries for "smell loss" (yellow) and "taste loss" (green).* ***C)*** *Online queries for "shortness of breath" (red curve) preceded the peak of the number of admissions to CCRU by three days.*

Supplementary Tables

Supplementary Table 1 : Data exclusions and Sampling strategy

	n
**Total GCCR participants**	41759
**Answering Smell change question**	18118
**Change in SMELL or TASTE**	15872
**Residents in France - ITALY - UK**	7929
**With date onset of first symptom**	6784
**FIGURE 1 :**	
French resident with onset of first symptom between 03-01-2020 and 05-11-2020	3832
France resident with information regarding the onset of Smell/taste change	1476
**FIGURE 2 :**	
FRANCE With date onset of first symptom between 02-01-2020 and 05-11_2020	**4720**
ITALY With date onset of first symptom between 02-01-2020 and 05-11_2020	**1241**
UK With date onset of first symptom between 02-01-2020 and 05-11_2020	**750**
FRANCE with date onset of SMELL/TASTE change between 02-01-2020 and 05-11_2020	**1487**
ITALY with date onset of SMELL/TASTE change between 02-01-2020 and 05-11_2020	**264**
UK with date onset of SMELL/TASTE change between 02-01-2020 and 05-11_2020	**243**

Supplementary Table 2*: Test of associations between putative indicators*

Test for association between paired samples, using one of Pearson's product moment correlation coefficient, without correction for multiple comparisons.(IDF= Ile de France, capital region fo France, "GCCR participants" =participant to the present study)

variableX	variableY	p	cor	type	level
proportion of GCCR participants without medical diagnostic	ratioER	7.32e-01	0,04	without IDF an	departement
proportion of GCCR participants without medical diagnostic	ratioER	7.54e-01	0,07	without IDF an	region
proportion of GCCR participants	ratioER	1.21e-01	0,17	without IDF an	departement
proportion of GCCR participants	ratioER	4.13e-01	0,19	without IDF an	region
proportion of GCCR participants without medical diagnostic	hospital death	1.91e-02	0,25	without IDF an	departement
proportion of GCCR participants without medical diagnostic	hospitalization	1.3e-02	0,27	without IDF an	departement
ratioER	CCRU admision	2.96e-03	0,32	without IDF an	departement
ratioER	hospitalization	1.16e-03	0,34	without IDF an	departement
ratioER	hospital death	1.05e-03	0,35	without IDF an	departement
courbatures_GOOGLE	ratioER	3.2e-01	0,38	without IDF an	region
proportion of GCCR participants without medical diagnostic	ratioER	8.01e-05	0,39	all	departement
proportion of GCCR participants	hospital death	6.42e-05	0,42	without IDF an	departement
proportion of GCCR participants	ratioER	1.6e-05	0,42	all	departement
proportion of GCCR participants without medical diagnostic	hospital death	5.58e-02	0,43	without IDF an	region
ratioER	CCRU admision	6.06e-02	0,43	without IDF an	region
courbatures_GOOGLE	ratioER	2.12e-01	0,43	all	region
ratioER	hospital death	5.02e-02	0,44	without IDF an	region
proportion of GCCR participants without medical diagnostic	hospitalization	4.73e-02	0,45	without IDF an	region
proportion of GCCR participants without medical diagnostic	ratioER	3.43e-02	0,45	all	region
proportion of GCCR participants without medical diagnostic	hospital death	3.19e-06	0,46	all	departement
ratioER	hospitalization	4.09e-02	0,46	without IDF an	region
courbatures_GOOGLE	CCRU admision	2.16e-01	0,46	without IDF an	region
proportion of GCCR participants	hospitalization	5.3e-06	0,47	without IDF an	departement
proportion of GCCR participants without medical diagnostic	CCRU admision	4.55e-06	0,47	without IDF an	departement
courbatures_GOOGLE	hospitalization	1.97e-01	0,47	without IDF an	region
proportion of GCCR participants	ratioER	2.23e-02	0,48	all	region
courbatures_GOOGLE	hospital death	1.86e-01	0,48	without IDF an	region
proportion of GCCR participants	hospital death	1.86e-07	0,5	all	departement
proportion of GCCR participants without medical diagnostic	hospitalization	2.49e-07	0,5	all	departement
ratioER	hospital death	1.86e-07	0,5	all	departement
ratioER	hospitalization	1.14e-07	0,51	all	departement
ratioER	CCRU admision	6.9e-08	0,52	all	departement
proportion of GCCR participants	hospitalization	6.08e-09	0,55	all	departement
proportion of GCCR participants without medical diagnostic	CCRU admision	1.21e-02	0,55	without IDF an	region
ratioER	CCRU admision	4.82e-03	0,58	all	region
ratioER	hospital death	4.97e-03	0,58	all	region
perte gout_GOOGLE	ratioER	8.1e-02	0,58	all	region
perte gout_GOOGLE	ratioER	1.01e-01	0,58	without IDF an	region
perte odorat_GOOGLE	hospital death	9.79e-02	0,59	without IDF an	region
ratioER	hospitalization	3.35e-03	0,6	all	region
perte odorat_GOOGLE	CCRU admision	8.91e-02	0,6	without IDF an	region
proportion of GCCR participants	hospital death	3.16e-03	0,63	without IDF an	region
fievre_GOOGLE	hospital death	7.15e-02	0,63	without IDF an	region
proportion of GCCR participants	CCRU admision	2.56e-11	0,64	without IDF an	departement
perte odorat_GOOGLE	hospitalization	5.52e-02	0,66	without IDF an	region
courbatures_GOOGLE	hospitalization	3.4e-02	0,67	all	region
courbatures_GOOGLE	CCRU admision	3.45e-02	0,67	all	region
courbatures_GOOGLE	hospital death	3.51e-02	0,67	all	region
fievre_GOOGLE	CCRU admision	4.96e-02	0,67	without IDF an	region
toux_GOOGLE	ratioER	4.88e-02	0,67	without IDF an	region
proportion of GCCR participants	hospitalization	1.03e-03	0,68	without IDF an	region
proportion of GCCR participants without medical diagnostic	hospital death	3.9e-04	0,69	all	region
toux_GOOGLE	ratioER	2.71e-02	0,69	all	region
fievre_GOOGLE	hospitalization	3.58e-02	0,7	without IDF an	region
fievre_GOOGLE	CCRU admision	2.43e-02	0,7	all	region
fievre_GOOGLE	hospital death	2.44e-02	0,7	all	region
proportion of GCCR participants without medical diagnostic	CCRU admision	1.69e-16	0,72	all	departement
proportion of GCCR participants	CCRU admision	3.02e-04	0,72	without IDF an	region
proportion of GCCR participants without medical diagnostic	hospitalization	8.13e-05	0,74	all	region
fievre_GOOGLE	hospitalization	1.43e-02	0,74	all	region
proportion of GCCR participants	hospital death	5.62e-05	0,75	all	region
proportion of GCCR participants	CCRU admision	4.16e-19	0,76	all	departement
toux_GOOGLE	CCRU admision	1.08e-02	0,76	all	region
perte gout_GOOGLE	CCRU admision	1.42e-02	0,78	without IDF an	region
perte odorat_GOOGLE	hospital death	8.23e-03	0,78	all	region
perte gout_GOOGLE	hospital death	1.12e-02	0,79	without IDF an	region
perte odorat_GOOGLE	ratioER	5.43e-03	0,8	all	region
proportion of GCCR participants	hospitalization	5.71e-06	0,81	all	region
perte odorat_GOOGLE	CCRU admision	3.97e-03	0,82	all	region
toux_GOOGLE	CCRU admision	7.2e-03	0,82	without IDF an	region
proportion of GCCR participants without medical diagnostic	CCRU admision	1.65e-06	0,83	all	region
perte odorat_GOOGLE	hospitalization	3.07e-03	0,83	all	region
toux_GOOGLE	hospital death	2.7e-03	0,83	all	region
toux_GOOGLE	hospitalization	2.62e-03	0,84	all	region
perte gout_GOOGLE	hospitalization	3.78e-03	0,85	without IDF an	region
toux_GOOGLE	hospital death	4.01e-03	0,85	without IDF an	region
proportion of GCCR participants	CCRU admision	8.95e-08	0,88	all	region
perte gout_GOOGLE	CCRU admision	7.66e-04	0,88	all	region
perte gout_GOOGLE	hospital death	7.31e-04	0,88	all	region
toux_GOOGLE	hospitalization	1.85e-03	0,88	without IDF an	region
fievre_GOOGLE	ratioER	4.55e-04	0,9	all	region
fievre_GOOGLE	ratioER	8.67e-04	0,9	without IDF an	region
perte gout_GOOGLE	hospitalization	1.74e-04	0,92	all	region
perte odorat_GOOGLE	ratioER	3.56e-04	0,92	without IDF an	region

Supplementary Table 3: *National and regional media coverage of the GCCR study in France*

type	medium	distribution	date	link
presse	BFM TV	national	14-avr	· https://www.bfmtv.com/sante/coronavirus-une-etude-internationale-lancee-
presse	orange	national	14-avr	· https://actu.orange.fr/france/lancement-d-une-vaste-enquete-sur-la-perte-
presse	msn	national	14-avr	· https://www.msn.com/fr-fr/actualite/coronavirus/coronavirus-une-
presse	BFM / RMC	national	14-avr	https://rmc.bfmtv.com/emission/un-questionnaire-en-ligne-pour-participer-a-
presse	femme actuel	national	14-avr	https://www.femmeactuelle.fr/sante/news-sante/coronavirus-comment-
presse	hufftingtonpos	national	14-avr	· https://www.huffingtonpost.fr/entry/vous-avez-eu-le-coronavirus-aidez-les-
presse	france info	national	14-avr	· https://www.francetvinfo.fr/sante/maladie/coronavirus/coronavirus-une-
presse	20 minutes	national	14-avr	· https://www.20minutes.fr/sante/2760607-20200414-coronavirus-enquete-
presse	nouvel obs	national	14-avr	· https://www.nouvelobs.com/sante/20200414.OBS27524/coronavirus-perte-
presse	le Figaro	national	14-avr	· https://www.lefigaro.fr/flash-actu/covid-19-enquete-internationale-sur-la-
presse	CNRS	national	14-avr	http://www.cnrs.fr/fr/covid-19-lancement-dune-enquete-internationale-sur-la-
presse	libération	national	14-avr	https://www.liberation.fr/direct/element/une-enquete-internationale-lancee-
blog	medisite	national	14-avr	https://www.medisite.fr/coronavirus-covid-19-la-perte-du-gout-et-de-lodorat-
blog	1001 infos	national	14-avr	· https://1001infos.net/france/coronavirus-une-enquete-internationale-lancee-
blog	la gazette du	national	14-avr	https://www.gazettelabo.fr/breves/9706Covid19-enquete-internationale-
blog	myscience	national	14-avr	https://www.myscience.fr/news/wire/covid_19_lancement_d_une_enquete_i
blog	yahoo sport	national	14-avr	https://fr.sports.yahoo.com/news/coronavirus-enqu%C3%AAte-scientifique-
blog	le blob	national	14-avr	· https://leblob.fr/actualites/covid-19-une-enquete-mondiale-pour-decrire-
blog	lactu24	national	14-avr	https://lactu24.com/26376-2/
radio	radio scoop	Auvergne / Rh	14-avr	https://radioscoop.com/infos/participez-a-une-enquete-sur-la-perte-de-l-
presse	lyonmag	Rhône-Alpes	14-avr	https://www.lyonmag.com/article/106956/coronavirus-une-etude-sur-la-perte-
radio	impact fm	Rhône-Alpes	14-avr	https://www.impactfm.fr/infos/participez-a-une-enquete-sur-la-perte-de-l-
presse	lyon capitale	Rhône-Alpes	14-avr	https://www.lyoncapitale.fr/actualite/coronavirus-lyon-participe-a-une-
presse	la depeche du	Languedoc-Ro	14-avr	https://www.ladepeche.fr/2020/04/14/covid-19-enquete-internationale-sur-la-
presse	golfe de St Tr	PACA	14-avr	https://golfedesainttropez.maville.com/actu/actudet_-coronavirus.-une-
presse	ma ville dragu	PACA	14-avr	https://draguignan.maville.com/actu/actudet_-coronavirus.-une-enquete-
presse	brignoles - ma	PACA	14-avr	https://brignoles.maville.com/actu/actudet_-coronavirus.-une-enquete-
presse	la provence	PACA	14-avr	https://www.laprovence.com/article/sante/5961521/covid-19-enquete-
blog	cres PACA	PACA	14-avr	http://www.cres-
presse	ouest france	Bretagne / Ce	14-avr	· https://www.ouest-france.fr/sante/virus/coronavirus/coronavirus-une-
presse	sante magazir	national	15-avr	· https://www.santemagazine.fr/actualites/actualites-sante/coronavirus-une-
presse	notre temps	national	15-avr	· https://www.notretemps.com/sante/covid-19-enquete-internationale-sur-la-
presse	latribune	national	15-avr	· https://www.latribune.ca/covid-19/participants-recherches-pour-une-
presse	science et ave	national	15-avr	https://www.sciencesetavenir.fr/sante/coronavirus-le-point-du-jour-sur-le-
blog	technoscience	national	15-avr	· https://www.techno-science.net/actualite/covid-19-lancement-enquete-
blog	presse19	national	15-avr	http://presse19.centerblog.net/4098-etude-internationale-sur-la-perte-de-
radio	tonic radio	Rhône-Alpes	15-avr	https://www.tonicradio.fr/covid-19-une-etude-lancee-face-a-la-perte-dodorat-
presse	centre jacques	Rhône-Alpes	15-avr	https://centrejacquescartier.com/actualites/detail/actu/covid-19-traitements-
presse	france region	Rhône-Alpes	15-avr	https://france3-regions.francetvinfo.fr/auvergne-rhone-alpes/coronavirus-
radio	KISS FM	PACA	15-avr	· http://www.kissfm.fr/page.asp?id=90
presse	france 3 PACA	PACA	15-avr	· https://france3-regions.francetvinfo.fr/provence-alpes-cote-d-
TV	france 3 PACA	PACA	15-avr	https://france3-regions.francetvinfo.fr/provence-alpes-cote-d-
presse	futurasciences	national	16-avr	· https://www.futura-sciences.com/sante/breves/coronavirus-covid-19-
presse	yahoo	national	16-avr	· https://fr.news.yahoo.com/covid-19-participez-%C3%A0-enqu%C3%AAte-
blog	anosmie.org	national	16-avr	https://www.anosmie.org/2020/04/16/enquetes-publiques-etudes-cliniques-
presse	Le Monde	national	20-avr	https://www.lemonde.fr/sciences/article/2020/04/20/combien-de-cas-de-
radio	France inter	national	17-avr	· https://www.franceinter.fr/environnement/detox-la-perte-du-gout-et-de-l-
radio	RCF	national	17-avr	https://rcf.fr/la-matinale/trois-questions-jerome-golebiowski
blog	pourquoi docte	national	17-avr	· https://www.pourquoidocteur.fr/Articles/Question-d-actu/32168-Coronavirus-
radio	RFI	national	20-avr	http://www.rfi.fr/ru/%D1%84%D1%80%D0%B0%D0%BD%D1%86%D0%B8
presse	top santé	national	20-avr	· https://www.topsante.com/medecine/maladies-
presse	inrae	national	20-avr	https://www.inrae.fr/covid-19
blog	nez - la revue	national	21-avr	https://www.nez-larevue.fr/magazine/actualites/covid-19-le-jour-ou-le-monde-
presse	UCA	PACA	21-avr	http://univ-cotedazur.fr/contenus-riches/actualites/fr/covid-19-lancement-
blog	breakingnews	national	22-avr	https://www.breakingnews.fr/sante/avez-vous-perdu-lodeur-ou-le-gout-des-
blog	seronet	national	23-avr	https://seronet.info/breve/enquete-sur-la-perte-de-lodorat-et-du-gout-87429
presse	actu toulouse	Midi-Pyrénées	23-avr	https://actu.fr/occitanie/toulouse_31555/toulouse-perte-gout-de-lodorat-
presse	france3 region	Languedoc-Ro	27-avr	https://france3-regions.francetvinfo.fr/occitanie/haute-
blog	CPU	national	02-mai	http://www.cpu.fr/actualite/covid-19-la-piste-du-gout-et-de-lodorat/
presse	tribune de lyo	Rhône-Alpes	03-mai	https://tribunedelyon.fr/salade-lyonnaise/article/sommeil-et-confinement-
presse	nice matin	PACA	03-mai	https://www.pressreader.com/france/nice-matin-nice-littoral-et-
presse	doctissimo	national	?	· https://www.doctissimo.fr/sante/news/coronavirus-enquete-internationale-
blog	crumpe	national	?	· https://www.crumpe.com/2020/04/une-enquete-scientifique-internationale-
presse	cnrs occitanie	Languedoc-Ro	?	https://www.cnrs.fr/occitanie-ouest/actualites/article/alerte-presse-covid-19-
blog	alternatif bien	national	?	https://alternatif-bien-etre.com/urgence-sante-coronavirus/des-chercheurs-
presse	univ lyon	Rhône-Alpes	?	https://popsciences.universite-lyon.fr/ressources/covid-19-odorat-et-qualite-
presse	cnrs centre es	Alsace / Champagne-Ardenn		http://www.dr6.cnrs.fr/centre-est/spip.php?rubrique26
presse	cnrs bretagne	Bretagne / Pa	?	https://www.dr17.cnrs.fr/
presse	cnrs aquitaine	Aquitaine	?	http://www.cnrs.fr/aquitaine/
presse	SCF	national	?	https://www.societechimiquedefrance.fr/Le-milieu-academique-se-mobilise-
