# Supplementary material for: Smell and taste changes are early indicators of the COVID-19 pandemic and political decision effectiveness
Source: Nat Commun. 2020 Oct 14;11:5152. doi: 10.1038/s41467-020-18963-y (PMC7560893; doi:10.1038/s41467-020-18963-y)
Supplement: Supplementary file 3 — Reporting Summary [file 41467_2020_18963_MOESM3_ESM.pdf]

# nature research

Corresponding author(s): Denis Pierron
Jerome Golebiowski
Moustafa Bensafi

Last updated by author(s): Aug 5, 2020

# Reporting Summary

Nature Research wishes to improve the reproducibility of the work that we publish. This form provides structure for consistency and transparency in reporting. For further information on Nature Research policies, seeAuthors & Referees and theEditorial Policy Checklist .

## Statistics

For all statistical analyses, confirm that the following items are present in the figure legend, table legend, main text, or Methods section.

| n/a | Confirmed | |
|---|---|---|
| [ ] | [x] | The exact sample size (*n*) for each experimental group/condition, given as a discrete number and unit of measurement |
| [ ] | [x] | A statement on whether measurements were taken from distinct samples or whether the same sample was measured repeatedly |
| [ ] | [x] | The statistical test(s) used AND whether they are one- or two-sided<br>*Only common tests should be described solely by name; describe more complex techniques in the Methods section.* |
| [ ] | [x] | A description of all covariates tested |
| [ ] | [x] | A description of any assumptions or corrections, such as tests of normality and adjustment for multiple comparisons |
| [ ] | [x] | A full description of the statistical parameters including central tendency (e.g. means) or other basic estimates (e.g. regression coefficient) AND variation (e.g. standard deviation) or associated estimates of uncertainty (e.g. confidence intervals) |
| [ ] | [x] | For null hypothesis testing, the test statistic (e.g. *F*, *t*, *r*) with confidence intervals, effect sizes, degrees of freedom and *P* value noted<br>*Give P values as exact values whenever suitable.* |
| [x] | [ ] | For Bayesian analysis, information on the choice of priors and Markov chain Monte Carlo settings |
| [x] | [ ] | For hierarchical and complex designs, identification of the appropriate level for tests and full reporting of outcomes |
| [ ] | [x] | Estimates of effect sizes (e.g. Cohen's *d*, Pearson's *r*), indicating how they were calculated |

*Our web collection on statistics for biologists contains articles on many of the points above.*

## Software and code

Policy information about availability of computer code

| | |
|---|---|
| Data collection | data are collected using these web-based surveys, https://gcchemosensr.org https://form.crnl.fr/index.php/146862?newtest=Y&lang=fr (limesurvey) |
| Data analysis | Google trends, R version 4.0.0 (2020-04-24) -- "Arbor Day" GUI 1.71 Catalina build (7827),mac-os catalina (10.15.4), custom R script are available on https://osf.io/gew7p/ |

For manuscripts utilizing custom algorithms or software that are central to the research but not yet described in published literature, software must be made available to editors/reviewers. We strongly encourage code deposition in a community repository (e.g. GitHub). See the Nature Research guidelines for submitting code & software for further information.

## Data

Policy information about availability of data

All manuscripts must include a data availability statement. This statement should provide the following information, where applicable:

- Accession codes, unique identifiers, or web links for publicly available datasets
- A list of figures that have associated raw data
- A description of any restrictions on data availability

Generated data are provided with this paper (Supplementary File).
Other dataset used are available on internet: pollens : https://pollens.fr; INSEE : https://www.insee.fr/fr/statistiques/1893198 ; OXFORD COVID https://www.bsg.ox.ac.uk/research/research-projects/coronavirus-government-response-tracker, centee publique france : https://geodes.santepubliquefrance.fr/#c=home, ECDC https://www.ecdc.europa.eu/en

# Field-specific reporting

Please select the one below that is the best fit for your research. If you are not sure, read the appropriate sections before making your selection.

☐ Life sciences ☒ Behavioural & social sciences ☐ Ecological, evolutionary & environmental sciences

For a reference copy of the document with all sections, see nature.com/documents/nr-reporting-summary-flat.pdf

# Behavioural & social sciences study design

All studies must disclose on these points even when the disclosure is negative.

**Study description**

In response to the COVID-19, many governments have taken unprecedented measures in peacetime, to avoid an overflow of intensive care units and critical care resuscitation units (CCRUs). Due to the heavy societal and economic impact of measure such as the lockdown, accurate means to characterize the spread of the disease would be extremely helpful for the reopening strategies. Concurrently, smell and taste changes have been identified as among the most specific symptoms of COVID-19.
Here, we show that self-reports of smell/taste changes are more closely associated with hospital overload and are much earlier than the current governmental indicators. We also show a decrease of new onset as early as 5 days after the lockdown enforcement, which is consistent with a rapid effect of the lockdown on the pandemic. Cross-country comparisons show countries with the most stringent lockdown measures (France and Italy) present a faster decline in new reports of the onset of smell/taste changes after the lockdown than a country with less stringent measures (United Kingdom).

Study type/design: Observational study, Quantitative cross-sectional

**Research sample**

Participants were recruited by word of mouth as well as through social and traditional media (flyers, social media, television, radio) during the COVID-19 pandemic. It was well covered by the French press, as over 70 articles mentioned the project, at both the regional and national level. Respondents received no monetary incentive for their participation.

In order to broadly investigate chemosensory disorders in France during the COVIS-19 crisis we allowed questionnaire completion only to participants who indicated they had suffered from a respiratory disease in the past two weeks, whether they noticed a change in their taste/smell or not.

Consequently, the number of responses analyzed in France was between n=1476 and 4720 depending on the analysis conducted (i.e., on whether the information of interest was present or missing and the date range of analysis. For comparison purposes, n=264 to 1241 participants from Italy and n=243 to 750 participants from the UK were included. The majority of the participants were women (FR:66.38%, IT:69.3%, UK:76.0%), and the average age was around 40 [FR=40.7(sd=12.4), IT=41.1(sd=11.4), UK=41.09(sd=12.1)]. In the French dataset, a total of 15% of individuals tested positive for COVID-19 (lab result) and 44% were clinically diagnosed by a practitioner based on their symptoms. The remaining 41% were not diagnosed for COVID-19 but declared a change in perception of either smell or taste.

When considering the present data, at least 3 parameters may contribute to a selection bias in our sample: (1) the age, (2) the gender of the participants, and (3) the format and the advertising of the survey. Regarding participant' age, our study cohort (mean 40.7 years, sd =12.4)) was quite similar to the French population mean (41.1 years22); however, we did only include individuals over 18 due to issues of consent, and administrative reasons, and seniors were also less represented. For gender, our sample contained a greater proportion of women (67%) compared to men, which might influence the results. However, additional analysis showed no differences in peaks of smell/taste changes across ages or gender, minimizing concerns that such selection biases may have influenced present results (See Supplementary Figure 2). We also tested the potential selection bias due to format and the advertising of the survey, by comparing the GCCR dataset with an independent second study performed on French residents (see Methods). Remarkably we observed highly similar results across studies where advertising, inclusion criteria, and survey format were different.

**Sampling strategy**

This study is mainly based on data from the Global Consortium for Chemosensory Research survey (GCCR)23 – a global, crowd-sourced online study deployed in 30+ languages24. The data analyzed here were collected from April 7 to May 14, 2020

Inclusion criteria were as follows. i) Questionnaire completion was allowed only to participants who indicated they had suffered from a respiratory disease in the past two weeks, whether they noticed a change in their taste/smell or not. ii) Participants aged 18 years old or younger were excluded.

The correlation analyses relate the number of self-reported smell and/or taste change in each state (“Région”) and parameters of overwhelmed hospitals (number of COVID-19+ cases, hospitalizations, reanimations, mortality cases due to COVID-19) within the same period of time. Overall, no appropriate effect size estimates exist for this study, because to our knowledge no studies exist that compare the number of participant of a questionnaire and the number of COVID-19+ cases, hospitalizations, reanimations, mortality cases due to COVID-19.
However, the only study which resembles to our is the one from Bagheri et al. (2020) who correlated the number of declared anosmia with the number of COVID cases in each of the 31 provinces of Iran; their sample size was 10069 individuals (average: 324 participants/ province). A power analysis of 80% is associated with a pearson correlation of 0.63 with 22 data-points (states). As France includes 22 states (now 13, but it’s better to consider the 2016 classification since the organization of the health system in France is based on this 22

| | |
|---|---|
| | state structure) |
| Data collection | Data are based on a global, crowd-sourced online study deployed in 30+ languages as well as one independant online survey restircted to France. Participants responded via an online web-based interface without the presence of researcher. |
| Timing | The data analyzed here represent the collection from April 7 to May 14 2020. |
| Data exclusions | Only participants above 18 years old were included, depending analysis participants are included based on location, gender, symptoms, detailed number are provided in supplementary table 1. In summary :<br>Answering Smell change question (exclusion = 23641), Change in SMELL or TASTE (exclusion = 2246), Residents in France - ITALY - UK (exclusion = 7943), With date onset of first symptom (exclusion = 1145)<br><br>For the analyses conducted in this article, only individuals reporting a change in smell and/or taste perception were included, based on the question "Have you had any of the following symptoms with your recent respiratory illness or diagnosis?". Moreover, to exclude unreliable entries, participants must have reported a quantitative difference of at least 5 on a 0-to-100 rating scale between their ability to smell and/or taste before and during their recent respiratory illness or diagnosis. We then extracted from full dataset individuals who reported living in France, Italy or the UK. |
| Non-participation | Regarding, the international online survey 41759 questionnaire have been started but only 18118 participants answered to the first question regarding their change of smell/taste. ( and only 15872 participant stating a change). In other words, 25887 participants were excluded (no respiratory disorder or smell/taste change) or dropped out. |
| Randomization | This is an observational study participant were not allocated to experimental groups. The potential sampling bias due to a link between the regional media coverage of our survey and the associated self-reported chemosensory changes by region was ruled out after confirming that these two variables were not correlated ($R < 0.01$, $p > 0.9$). |

# Reporting for specific materials, systems and methods

We require information from authors about some types of materials, experimental systems and methods used in many studies. Here, indicate whether each material, system or method listed is relevant to your study. If you are not sure if a list item applies to your research, read the appropriate section before selecting a response.

## Materials & experimental systems

| n/a | Involved in the study | |
|---|---|---|
| ☒ | ☐ | Antibodies |
| ☒ | ☐ | Eukaryotic cell lines |
| ☒ | ☐ | Palaeontology |
| ☒ | ☐ | Animals and other organisms |
| ☐ | ☒ | Human research participants |
| ☒ | ☐ | Clinical data |

## Methods

| n/a | Involved in the study | |
|---|---|---|
| ☒ | ☐ | ChIP-seq |
| ☒ | ☐ | Flow cytometry |
| ☒ | ☐ | MRI-based neuroimaging |

# Human research participants

Policy information about studies involving human research participants

| | |
|---|---|
| Population characteristics | see above |
| Recruitment | Participants were recruited by word of mouth as well as through social and traditional media (flyers, social media, television, radio) during the COVID-19 pandemic. The potential sampling bias due to a link between the regional media coverage of our survey and the associated self-reported chemosensory changes by region was ruled out after confirming that these two variables were not correlated ($R < 0.01$, $p > 0.9$). . All recent COVID-19 surveys, including our own, suffer from biases in the profile of respondents, as they may be more willing and interested in participating in scientific research. |
| Ethics oversight | The protocol complies with the revised Declaration of Helsinki and the international project was approved as an exempt study by the Office of Research Protections at The Pennsylvania Study University (Penn State) in the U.S.A. (STUDY00014904). French survey was approved by the CNRS ethics committee.This anonymous study was conducted according to the French ethical legislation on human experimentation via questionnaire and was validated by the CNRS National Institute for Biological Sciences. |

Note that full information on the approval of the study protocol must also be provided in the manuscript.
